# Interleukin-15 and chemokine ligand 19 enhance cytotoxic effects of chimeric antigen receptor T cells using zebrafish xenograft model of gastric cancer

**DOI:** 10.3389/fimmu.2022.1002361

**Published:** 2022-12-23

**Authors:** Zhifeng Zhou, Jieyu Li, Jingwen Hong, Shuping Chen, Mingshui Chen, Ling Wang, Wansong Lin, Yunbin Ye

**Affiliations:** ^1^ Laboratory of Immuno-Oncology, Clinical Oncology School of Fujian Medical University, Fujian Cancer Hospital, Fuzhou, Fujian, China; ^2^ Fujian Key Laboratory of Translational Cancer Medicine, Fuzhou, Fujian, China; ^3^ School of Basic Medical Sciences, Fujian Medical University, Fuzhu, Fujian, China

**Keywords:** gastric cancer, NKG2D (natural killer group 2 member D), CAR-T cells, IL-15, CCL19

## Abstract

Chimeric antigen receptor (CAR) T cells have been proven effective for the treatment of B-cell-mediated malignancies. Currently, the development of efficient tools that supply CAR T cells for the treatment of other malignancies would have great impact. In this study, interleukin (IL)-15 and C-C motif chemokine ligand 19 (CCL19) were introduced into natural killer group 2D (NKG2D)-based CARs to generate 15×19 CAR T cells, which remarkably increased T-cell expansion and promoted the production of central memory T (T_cm_) cells. 15×19 CAR T cells showed greater cytotoxicity to gastric cell lines than conventional CAR T cells and produced higher levels of IL-15 and CCL-19, which resulted in increased responder T cell chemotaxis and reduced expression of T cell exhaustion markers. A live zebrafish model was used for single-cell visualization of local cytotoxicity and metastatic cancers. Administration of 15×19 CAR T cells resulted in significant shrinking of gastric cancer xenograft tumors and expansion of 15×19 CAR T cells in zebrafish models. Taken together, these findings demonstrate that 15×19 CAR T cells are highly efficient in killing gastric cancer cells, are effective to avoid off-target effects, and migrate to local and metastatic sites for long-term surveillance of cancers.

## Introduction

Gastric cancer is the fifth most common type of cancer and the fourth leading cause of cancer-related mortality worldwide (1). In 2020, there were approximately 1.09 million newly diagnosed cases of gastric cancer and more than 769 thousand people worldwide died due to this malignancy (2). Currently, treatments for gastric cancer primarily include gastrectomy, chemotherapy, radiotherapy, and targeted immunotherapy (3–5). Prognosis for this common malignancy depends on the stage at which it is diagnosed (1). The five-year survival rate in early-stage patients is over 90%, but the prognosis remains very poor in patients with advanced, unresectable, or metastatic disease (6). The development of highly effective, safe, and well-tolerated treatments for gastric cancer is a high priority for many researchers and clinicians (4).

Chimeric antigen receptor (CAR) T cells are typically generated from a single-chain variable fragment (scFv) of a monoclonal antibody (mAb) and a CD3ζ subunit of the T cell receptor (TCR) (7). CAR T cells specifically recognize tumor cell surface antigens and activate lymphocytes *via* intracellular signal transduction to increase T cell targetability and activity, thereby enabling the same cell to kill multiple types of cancer cells (8). Previous clinical trials have shown that CAR T cell therapy achieves satisfactory outcomes for the treatment of hematological malignancies (9), and there is also evidence showing efficacy of CAR T cell therapy for gastrointestinal tumors (10, 11).

Currently, there are two important issues related to CAR T cell therapy for gastric cancer that limits its use. Firstly, due to the heterogeneity of target antigens in gastric cancer, the CAR T cells have low specificity that causes severe adverse effects, including off-target effects and cytokine storms (12). Secondly, physical and immune barriers generated by the matrix and immune cells surrounding the cancer prevent CAR T cells from completely infiltrating tumor tissues, and the hypoxia and lack of nutrients inside tumors reduces the long-term survival and expansion of CAR T cells (13, 14).

Two important mediators of T cell function and recruitment were used in the current study. Interleukin (IL)-15 has been identified as a main regulator of T cell homeostasis, which plays a critical role in T cell expansion, memory, survival, and persistence (15–17). C-C motif chemokine ligand (CCL) 19 is effective at recruiting T cells and dendritic cells (DCs) to infiltrate into cancer sites (18, 19). As such, the IL-15 cytokine and CCL19 chemokine were introduced into natural killer group 2D (NKG2D)-based CARs to generate novel NKG2D-based CAR T cells. Additionally, preclinical testing to improve the efficacy and safety of CAR T cells is commonly performed in mouse models. However, these animal models are high in cost, complex, and can be slow to obtain results. Additional preclinical models may help fill the gap from *in vitro* assays to xenotransplantation in mouse models. Therefore, in the current study CAR T cell-mediated cytotoxicity was assessed *in vivo* using a zebrafish xenotransplantation model.

## Materials and methods

### Cell lines

Human gastric cancer cell lines (AGS, HCG-27 and MKN-45) were purchased from the Cell Bank of Chinese Academy of Sciences (Shanghai, China) and incubated in RPMI 1640 medium (GIBCO; Grand Island, NY, USA) supplemented with 10% fetal bovine serum (FBS; PAN-Seratech GmbH, Aidenbach, Germany) and 1% penicillin/streptomycin (MP Biomedicals; Santa Ana, California, USA). Gastric cancer antigens were prepared by treating gastric cancer cells with mitomycin (20 μg/mL; Sigma-Aldrich Corporation; St. Louis, MO, USA) for 2 hours, followed by washing twice in Dulbecco’s phosphate-buffered saline (DPBS; GIBCO) to eliminate gastric cancer cell viability but retain antigenicity.

### CAR expression vectors and transduction into human T cells

Anti-human NKG2D scFv was prepared according to previous reports (20, 21) with modifications. The extracellular region of NKG2D (amino acids 82-216) connected the hinge structural domain of CD8α in the framework and was fused in tandem with the human CD28 transmembrane region, the CD28,4-1BB co-stimulatory signaling region and the intracellular activating domain of CD3ζ. The CAR construct sequences were synthesized and inserted into multiple cloning sites of pHBLV-CMV-MCS-EF1-ZsGreen (Hanbio; Shanghai, China), which expressed green fluorescent protein (GFP) to generate the lentiviral plasmid LV-CMV-CAR-EF1-ZsGreen. Following validation by sequencing, lentiviral packaging was performed. 293T cells were co-transfected with the psPAX2 (packaging) and pMD2G (viral fusion protein) plasmids. The supernatant was collected and stored at -80°C. To generate the novel 15×19 CARs, the phosphoglycerate kinase (PGK) promoter along with IL-15 and CCL19 were introduced into NKG2D-based CARs, and the 2A sequence was embedded between IL-15 and CCL19 to allow for separate expression of IL-15 and CCL19.

Human T cells were transduced with the CAR-encoding lentiviral vectors. Briefly, blood samples (approximately 10 mL) were collected with anticoagulants from healthy volunteers, and peripheral blood mononuclear cells (PBMCs) were separated using lymphocyte separation medium (MP Biomedicals). PBMCs were incubated in T-lymphocyte serum-free KBM581 medium (Corning, Inc.; NY, USA), then seeded onto 6-well plates (Corning, Inc.) at a density of 2 x 10^6^ cells/mL. Cells were then incubated with anti-human CD3 and CD28-coated microbeads (Miltenyi Biotec, Inc.; Auburn, CA, USA) as well as IL-2 (100 U/mL) at 37°C for 24 hr. The three treatment groups included the NT group (cells without lentivirus transfection), conventional CAR group (cells treated with conventional CAR T cells), and NKG2D 15×19 CAR group (cells treated with NKG2D 15×19 CAR T cells). Cells were then transduced with lentiviral vectors (Hanbio; Shanghai, China) at a multiplicity of infection (MOI) of 10:1 and incubated at 37°C for 24 hr. Subsequently, the supernatant was removed and incubated at 37°C. Following transduction, *in vitro* cell amplification was performed in the presence of IL-2. Solutions were added at a final concentration of 100 IU/mL every 2-3 days, and cell density was maintained at 1 x 10^6^ to 2 x 10^6^ cells/mL. The CAR T cells positive for both GFP and NKG2D were detected using flow cytometry until the specified cell dose was achieved.

### Flow cytometry

Cells were incubated with corresponding flow cytometry antibodies (5 μL) at room temperature for 15 min, washed twice in phosphate-buffered saline (PBS) and run on a FACSCanto II flow cytometry system (BD Biosciences; San Jose, CA, USA) with an isotype control (BioLegend; San Diego, CA, USA) for each assay. To detect the expression of NKG2D ligand (NKG2DL) in AGS, HCG-27, and MKN45 cells, the cells were stained with NKG2D/CD314 Fc Chimera (clone Pro100­Lys330; R&D Systems, Minneapolis, MN, USA) and specific monoclonal antibodies including anti-MHC class I chain-related protein A/B (MICA/B; clone D7; R&D Systems), anti-UL16 binding protein 1 (ULBP-1; clone 170,818; R&D Systems), anti-ULBP-3 (clones 166,510; R&D Systems), anti-ULBP-2/5/6 (clones 165,903; R&D Systems) and anti-ULBP-4 (clones 709,116; R&D Systems). To measure the multiplicity of cell expansion, 2× cell suspension was mixed with Cell Proliferation Dye eFluor™ 670 (10 μM; eBioscience, Inc.; San Diego, CA, USA) in PBS at a ratio of 1:1 and incubated at 37°C for 10 min in the dark. The reaction was terminated by adding 4-5× volume of cold complete media, followed by incubation on ice for 5 min. Cells were then washed three times in PBS with complete media, and cell expansion was measured once daily. To detect the efficiency of CAR T cell infection, the proportion of CAR T cells expressing both APC-NKG2D and GFP was measured using the anti-NKG2D monoclonal antibody (clone 1D11; BD Bioscience). Percentages of CAR T cells were detected in CD4^+^ and CD8^+^ T cells by staining with PerCP-labeled anti-CD3 (clone SP34-2 RUO; BD Bioscience) and R-phycoerythrin (PE)-labeled anti-CD3 monoclonal antibodies (clone UCHT1; BD Bioscience). Cells were stained with allophycocyanin (APC)-labeled anti-CD45RO (clone UCHL1; BD Bioscience) and PE-labeled anti-CD62L monoclonal antibodies (clone SK11; BD Bioscience) to detect the proportion of central memory T (T_cm_) cells. APC-labeled anti-annexin V (BioLegend) and PE-labeled anti-7-aminoactinomycin D (7AAD) antibodies (BioLegend) were used to detect apoptosis in CAR T cells. To detect CAR T cell activation, the cells were stained with PE-labeled anti-CD3 (clone UCHT1; BD Bioscience), PE-labeled anti-CD25 (clone 2A3; BD Bioscience) and PE-labeled anti-human leukocyte antigen (HLA)-DR monoclonal antibodies (clone L243; BD Bioscience). To detect the exhaustion of CAR T cells, PE-labeled anti-CD3 (clone UCHT1; BD Bioscience), PE-labeled anti-programmed cell death-1 (PD1; clone EH12.1; BD Bioscience) and PE-labeled anti-CTLA4 monoclonal antibodies (clone BNI3; BD Bioscience) were used.

### Transwell migration assay

To detect responder T cell chemotaxis mediated by 15×19 CAR T cells, the cells were stained with Cell Proliferation Dye eFluor™ 670, and responder T cells (200 μL) were placed in the upper Transwell chamber (Roche Applied Sciences; Basel, Switzerland). NT, CAR T, or 15×19 CAR T cells were co-cultured with mitomycin C-treated HCG-27 cells for 3 days, and these cells (200 μL) were plated onto the lower Transwell chamber for a 3 or 5 hr incubation period. The number of cells that had migrated to the lower chamber was detected using flow cytometry. In addition, cells were stained with anti-CD15 (BD Bioscience) and anti-CCR7 monoclonal antibodies (BD Bioscience) to block IL-15 and CCL19 expression, and the percentage of migrated cells was detected using flow cytometry.

### Real-time cell analysis

Approximately 50 µL of HGC-27 cells were seeded onto an E-plate PET 16 (Omni Life Science; Bremen, Germany) at a density of 2 × 10^5^ cells/mL per well, and incubated at room temperature for 30 min. The E-plate PET 16 was placed in the RTCA S16 Station system (Acea Bioscience; San Diego, CA, USA) and incubated at 37°C with 5% CO_2_ overnight. Then, the same volume of effector cells was added to each well to allow an effector to target cell ratio of 1:1, and cells were incubated at 37°C with 5% CO_2_ for at least 48 hr. Cell index (CI) values were recorded once every 10 min using the RTCA xCELLigence DP (Acea Bioscience), and the absolute (delta) CI values were estimated by normalizing the CI to the time points prior to the addition of effector cells. The dynamic monitoring of fluorescence intensity of target cells was performed in 24-well plates. Briefly, HCG-27 cells were stained with PKH26 (red) to allow for a final volume of 500 μL in each well. The effector and target cells were co-cultured at a ratio of 1:1, and the cytotoxicity of effector cells to tumor cells was detected 0, 4, and 24 hr post-incubation.

### Enzyme-linked immunospot assay

The number of interferon gamma (IFN-γ) spot-forming cells was measured using the ELISpot assay kit (Dakewe Biotech Co., Ltd.; Shenzhen, China) following the manufacturer’s instructions. Briefly, PBMCs from each of the three treatment groups (NT, conventional CAR and NKG2D 15×19 CAR T cells) served as the effector cells, and gastric cancer cells served as the target cells. The effector cells and target cells were co-cultured at a ratio of 5:1 at 37°C for 24 hr, and the number of IFN-γ spot-forming cells was counted using an AID EliSpot Reader classic (Autoimmun Diagnostika GmbH; Strassberg, Germany).

### Cytokine release assays

Effector cells were co-cultured with HCG-27 cells at a density of 5 x 10^4^ cells/well on 96-well plates in triplicate. KBM581 media was added to a final volume of 200 μL, and cells were incubated for 3 days, then centrifuged at 400 × *g* for 5 min. The supernatant was collected for determination of IFN-γ, IL-15 and CCL19 expression using enzyme-linked immunosorbent assays (ELISA; Biomatik; Ontario, Canada). The expression of IL-1, IL-4, IL-6, IL-10, tumor necrosis factor (TNF)-α and IL-17A was detected using a CytometricBead array (CBA) on a FACSCanto II flow cytometry system, and all data were managed using the FCAP Array™ software version 3.0 (BD BioSciences).

### Zebrafish xenograft assay

Zebrafish were purchased from Fuzhou Bio-Service Biotechnology Co. Ltd. (Fuzhou, China), and xenotransplantation was performed using GB100T-8P injection glass capillaries (Science Products GmbH; Hofheim am Taunus, Germany) pulled with FemtoJet 4i microinjectors (Eppendorf; Hamburg, Germany). HCG-27 cells were stained with a red-fluorescent lipophilic membrane dye (5 μM; 1,1’-dioctadecyl-3,3,3’,3’-tetramethylindocarbocyanine perchlorate; Dil; Meilun Biotechnology, Dalian, China), and injected into zebrafish 48 hr post-fertilization at a density of 200 cells per fish. Stained HCG-27 cells were stored at 35°C. Effector cells from four groups (untreated, NT, conventional CAR, and 15×19 CAR) were injected 24 hr post-injection with the same number of HCG-27 cells at the same site. Since cells in the NT group did not express fluorescence, cells were stained green prior to injection (using fluorescent lipophilic 3,3’-dioctadecyloxacarbocyanine perchlorate; DiO; Meilun Biotechnology). Since cells in the conventional CAR and NKG2D 15×19 CAR groups expressed GFP, no additional fluorescence staining was required. Zebrafish were then visualized under a fluorescent stereomicroscope (Nikon; Tokyo, Japan) at 0 and 24 hr post-injection (hpi), and the area of fluorescence in each fish was determined using ImageJ software.

To test the cytotoxicity of CAR T cells against gastric cancer *in situ*, HCG-27 cells and effector cells were injected into the vitellicle. To test the cytotoxicity of CAR T cells against metastatic tumors, HCG-27 cells and effector cells were injected into the perivitelline space.

### Immunofluorescence

Frozen slides were brought to room temperature, fixed in cold acetone for 10 min, then air dried. Samples were incubated in proteinase K working solution at 37°C for 25 min. Next, permeabilize working solution was used to cover objective tissue, then incubate at room temperature for 20 min. The slices were equilibrated at room temperature: The following kits and antibodies were used for staining: TUNEL Assay Kit (G1501, Servicebio, China), rabbit anti-KI67 antibody (1:200; GB121141, Servicebio, China), goat anti-CD3 antibody (1:200; GB111337, Servicebio, China), 488 anti-goat (1:400; GB25303, Servicebio, China) and Cy5 anti-rabbit (1:400; GB27303, Servicebio, China). The slices were washed three times with PBS (pH 7.4). Throwed away liquid slightly, then coverslip with anti-fade mounting medium. Images were acquired using an OLYMPUS laser scanning microscope.

### Statistical analyses

All statistical analyses were performed using GraphPad Prism software version 9.0. All measurement data were described as mean ± standard deviation (SD) and a *P* value < 0.05 was considered statistically significant.

## Results

### NKG2DL expression in gastric cancer cells

Human NKG2DLs contain eight types of ligands, including MHCA/B and ULBP 1 to 6 (22), and NKG2DLs are either absent or rarely expressed in normal cells. Upon infection or the development of cancer, a large increase in NKG2DLs expression can be detected in cells (23, 24).

**Figure 1:**
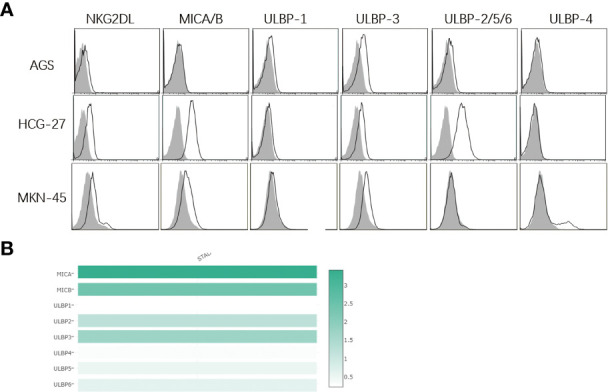
Expression of NKG2D ligands on surface of human gastric cancer cell lines. **(A)** Flow cytometry detects the expression of NKG2D ligands in human gastric cancer cell lines. The white histogram indicates expression of NKG2D-Fc, MICA/B, ULBP-1, ULBP-3, ULBP-2/5/6 and ULBP-4, and the shadow histogram indicates the expression of the matched isotype control. MICA/B is highly expressed in HCG-27 and MKN-45 cells, and moderate ULBP3 expression is detected in AGS, HCG-27 and MKN-45 cells, while low ULBP1/4 expression is found in AGS, HCG-27 and MKN-45 cells; **(B)** NKG2D ligand expression is analyzed in stomach adenocarcinoma in the Gepia server, and the highest MICA expression (score 3.4) is seen, followed by MICB expression (score 2.4), ULBP3 expression (score 1.7), ULBP2 (score 1.2), ULBP6 (score 0.6), ULBP5 (score 0.5), ULBP4 (score 0.3) and ULBP1 (score 0.2), which is almost in agreement with the expression of NKG2D ligands in human gastric cancer cell lines.

In the current study, the presence of NKG2DL expression on the surface of human gastric cancer cell lines was first detected. Flow cytometric analysis revealed that the recombinant human IgG1-Fc fusion protein (a NKG2D receptor) recognized all ligands of NKG2D receptors, and NKG2DL expression was detected in all three human gastric cancer cell lines (**Figure 1A** **)**. In addition, moderate ULBP-3 expression was found in AGS cells, and high MICA/B and ULBP-2/5/6 expression was detected in HCG-27 cells. In MKN-45 cells, moderate MICA/B, ULBP-3 and ULBP-4 expression was seen. These data demonstrate that NKG2DLs are widely expressed in human gastric cancer cell lines. Our previous study showed expression of one type of NKG2DLs in gastric cancer and non-small cell lung cancer (25, 26).

Next, data regarding NKG2DL expression in stomach adenocarcinoma was retrieved from the Gepia server (http://gepia.cancer-pku.cn/index.html). MICA/B had the highest expression levels, whereas ULBp2/3/5/6 expression was moderate and ULBp1/ULBP4 expression was the lowest, as presented in **Figure 1B**. Importantly, these data were similar to our findings.

### Construction and expression of NKG2D 15×19 CAR

**Figure 2:**
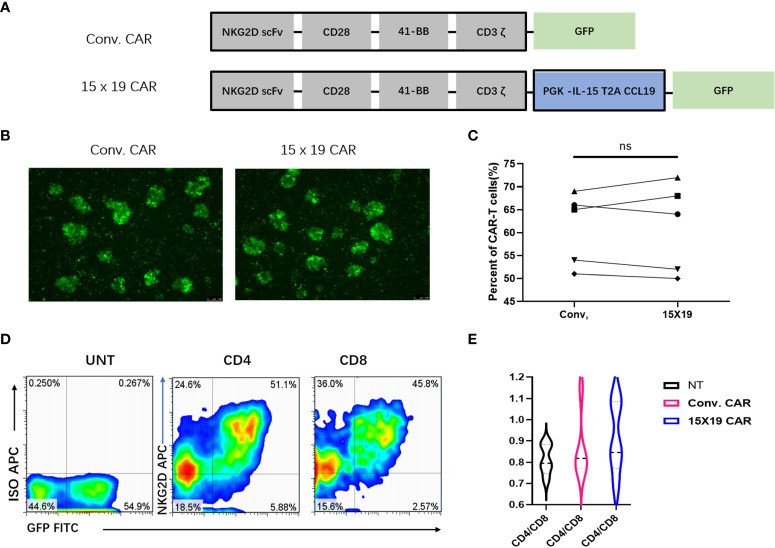
Generation of a specific CAR construct and *in vitro* expansion of CAR T cells. **(A)** The conventional CAR construct contains the extracellular region of human NKG2D receptor, which links with the transmembrane region and intracellular region of CD28, and the co-stimulatory domain of intracellular CD137 (4-1BB) and CD3ζ signal motif. The IL-15/CCL19 sequences-conjugated PGK promoter with 2A sequence embedding is introduced to conventional CARs to generate 15 × 19 CARs, which present the fluorescence of GFP; **(B)**, Green fluorescence is present in conventional CAR T cells and 15 × 19 CAR T cells 72 hour post-infection; **(C)**, No significant difference is seen in the cell transduction efficiency (both > 50%) between the conventional and 15 × 19 CAR groups. Analyzed for statistical significance by paired Student’s *t* test (*P* =0.8712); **(D)**, Expression of NKG2D (APC)-positive and GFP-positive CAR T cells in CD4^+^ and CD8^+^ T cells. Representative density plots are created from five independent experiments; **(E)**, There is no significant difference in the CD4/CD8 ratio among the three groups (*P* > 0.05). Data are captured from three independent healthy volunteers.

CARs expressed on T cells consist of an extracellular targeting domain derived from the antigen-binding site of an antibody, a transmembrane domain, and an intracellular signaling domain, to promote T cell activation elicited by an antigen or ligand. NKG2D CAR T cells target NKG2D ligands on cancer cells. In the current study, IL-15/CCL19-free anti-human NKG2D CARs containing CD28, 4-1BB and CD3ζ sequences (conventional CARs) were generated. Next, the IL-15/CCL19 sequences under the PGK promoter with 2A sequence embedding was added to conventional CARs to generate NKG2D 15×19 CARs. Diagrams of the engineered conventional CARs and 15×19 CARs are shown in **Figure 2A**.

Following transduction of CAR-encoding lentiviral vectors into human PBMCs for 72 hr, fluorescent microscopy revealed GFP fluorescence (**Figure 2B**). CAR T cells were positive for both GFP and NKG2D, and flow cytometry detected no significant difference in the positive rate of CAR T cells between conventional CARs and 15×19 CARs generated by T cells from the same donor (**Figure 2C**).

Next, the proportion of CAR in CD4^+^ and CD8^+^ T cells was detected, and the efficiencies of CD4^+^ and CD8^+^ cell transfection were approximately 51% and 45%, respectively (**Figure 2D**). These results implicate a highly efficient transfection of T cells by the designed CAR vector, which warranted the subsequent functional assays.

Activated CD8^+^ cells have been shown to have higher proliferative rates than activated CD4^+^ cells, which may promote the relative abundance of CD8^+^ cells in culture (27). At the start of the culture, 30% of the CD3^+^ cells were CD8^+^ T cells. The CD4/CD8 ratio was 0.8:1 in the 15×19 CAR T cells 7 days post-stimulation, which was not significantly different from other groups (*P* > 0.05) (**Figure 2E**). Therefore, no remarkable deviation in CD4/CD8 ratios or the presence of CD4^+^ cells facilitated increased antitumor effects *in vivo* (28).

The subsequent step was to examine proliferation, chemotaxis, and effector functions *in vitro* upon coculture of 15×19 CAR T cells with gastric cancer lines. However, the gastric cancer lines express MHC molecules and the T cells harbor a functional TCR. As such, the extent of the CAR-mediated effects on proliferation, expansion, migration, IFN-g release, and cytotoxicity are difficult to assess. Although non-CAR-mediated responses appear to be very moderate in the subset of T cells that were not transduced, the effects of MHC alone were assessed using a control group to detect the expression of MHC molecules in gastric cancer cell lines. HLA-A02 was found to be expressed in AGS cells, HLA-A24 in MKN-45 cells, and HLA-A24 in HCG-27 cells, while HLA-02 and HLA-A24 were not expressed in donor samples (**Supplementary Figure S1**), indicating completely mismatched MHC molecules. Although there was a complete MHC mismatch between target tumor cells and donor-derived T cells, the reactivity of NT T cells was low, suggesting low alloreactivity.

### *In vitro* phenotypic features of 15×19 CAR T cells

**Figure 3:**
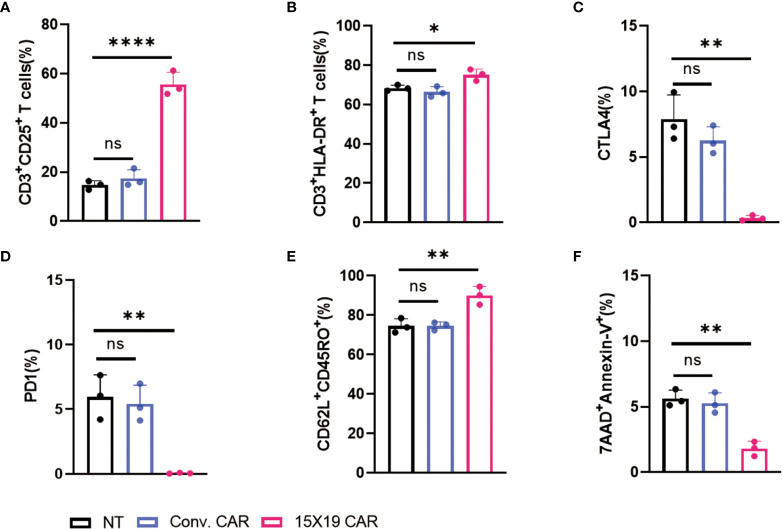
Phenotypic characterization of 15 × 19 CAR T cells. **(A)**, A higher proportion of CD3^+^CD25^+^ cells is detected in the 15 × 19 CAR group than in the NT and conventional CAR groups; **(B)**, A higher proportion of CD3^+^HLA-DR^+^ cells is detected in the 15 × 19 CAR group than in the NT and conventional CAR groups; **(C)** Lower CTLA4 expression is detected in the 15 × 19 CAR group than in the NT and conventional CAR groups; **(D)**, Lower PD1 expression is detected in the 15 × 19 CAR group than in the NT and conventional CAR groups; **(E)** A higher proportion of CD62L^+^CD45RO^+^ cells is detected in the 15 × 19 CAR group than in the NT and conventional CAR groups; **(F)** A lower apoptotic rate is seen in the 15 × 19 CAR group than in NT and conventional CAR groups. Data are presented as means ± SD from three independent experiments, and one-way ANOVA with Bonferroni’s correction is used for comparison of the percentage of phenotypic characterization. *ns*, no significant difference; *P < 0.05; **P < 0.01; ****P < 0.001.

Next, the phenotypic features of transfected CAR T cells were examined. Cells in the NT, conventional CAR, and 15×19 CAR groups were co-cultured with mitomycin C-treated HCG-27 cells at a ratio of 1:1 for 24 hr. Flow cytometry revealed increased CD25 and HLA-DR expression in the 15×19 CAR group compared to the NT and conventional CAR groups in mitomycin C-treated HCG-27 cells (**Figures 3A, B**). Previous studies have shown that the interaction between tumor cells and CAR T cells may trigger the expression of immunosuppressive factors (such as cytotoxic T-lymphocyte-associated antigen 4 [CTLA-4] and programmed cell death protein 1 [PD-1]) resulting in T-cell exhaustion or dysfunction. Importantly, the exhaustion of CAR T cells following long-term stimulation of tumor antigens is a factor that leads to disease relapse or drug resistance (29). Following stimulation of immune cells with tumor antigens, lower CTLA4 and PD1 expression was detected in the 15×19 CAR group compared to the NT and conventional CAR groups (**Figures 3C, D**).

In addition, a significantly higher proportion of T_cm_ cells (CD62L^+^CD45RO^+^ cells) and a lower rate of apoptotis were seen in the 15×19 CAR group compared to the NT and conventional CAR groups (**Figures 3E, F**). T_cm_ cells, which have self-renewal and replicating activity, have effective long-term *in vivo* antitumor actions due to long-term persistence (30, 31). These data demonstrate that 15×19 CAR T cells retain potent effector functions, up-regulate T_cm_ cell markers, and prevent T cell exhaustion.

### 15×19 CAR T cells promote T cell proliferation and migration

**Figure 4:**
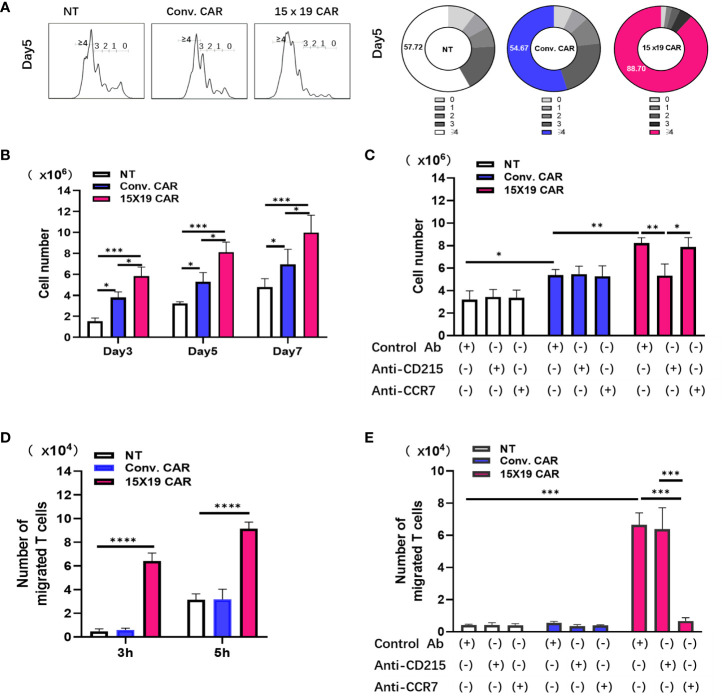
15 × 19 CAR T cells promote T cell proliferation and migration. **(A)** Cells in the NT, conventional CAR and 15 × 19 CAR groups are stained with Cell Proliferation Dye eFluor™ 670, co-cultured with mitomycin C-treated HCG-27 cells, and incubated for 3 days. Number of differentiated cells is shown in the histogram. Numbers in the donut charts represent the percentage of each gated fraction in the cell culture; **(B)**, The absolute numbers of survival cells in the NT, conventional CAR and 15 × 19 CAR groups at different time points; **(C)**,Antibody-blocking assay detects the effects of CD215 or CCR on cell proliferation. The absolute numbers of living cells are estimated after CD215 or CCR7 antibody blockade. *P = 0.0491 (NT with Control antibody vs. Conv. with Control antibody);**P = 0.0051 (Conv. with Control antibody vs. 15×19 with Control antibody); **P = 0.0046 (15×19 with Control antibody vs. 15×19 with CD215);*P = 0.0148 (15×19 with CD215 vs. 15×19 with CCR7); **(D)**, Chemotaxis of T cells by cells in the NT, conventional CAR and 15 × 19 CAR groups. ***P < 0.0001(3 h); ***P < 0.0001 (5 h); **(E)**, Antibody-blocking assay detects the effects of CD215 or CCR on T cellchemotaxis. The migrating cells are estimated after CD215 or CCR7 antibody blockade. ***P < 0.0001(NT and Conv. with Control antibody vs. 15×19 with Control antibody). ***P < 0.0001 (15×19 with Control antibody vs. 15×19 with anti-CCR7), ***P < 0.0001 (15×19 with anti-CD215 vs. 15×19 with anti-CCR7).All data are described as mean ± SD from three independent experiments. Analyzed for statistical significance by one-way ANOVA with multiple comparisons test.

Compared with IL-2 or IL-7, IL-15 was found to increase the persistence and efficacy of CAR T cells in multiple myeloma (32). In the current study, IL-15 was introduced to NKG2D CAR T cells, and the resulting 15×19 CAR T cells secreted high levels of IL-15 and massively expanded upon stimulation by tumor antigens. Cells in the NT, conventional CAR, and 15×19 CAR groups were labeled with Cell Proliferation Dye eFluor™ 670 and co-cultured for 3 days with mitomycin C-treated HCG-27 cells. Compared to the conventional CAR group, there were many more cells detected in the 15×19 CAR group (**Figures 4A, B**). Next, the NT, conventional CAR, and 15×19 CAR cells were co-cultured with mitomycin C-treated HCG-27 cells and incubated with an anti-CD215 (IL-15 receptor) antibody, an anti-CCR7 (CCL19 receptor) antibody, or an isotype control for 5 days. The enhanced expansion of 15×19 CAR T cells was completely attenuated by the addition of the anti-CD215 antibody, but not the anti-CCR7 antibody (**Figure 4C**), indicating that IL-15 was essential for the increase in 15×19 CAR T cell proliferation and survival.

Since CCL19 is a chemotactic agent of T cells and DCs, Transwell migration assays were performed to measure cell migration under a variety of conditions. Responder T cells stained with Cell Proliferation Dye eFluor™ 670 were placed in the upper Transwell chamber, whereas NT, conventional CAR, or 15×19 CAR cells stimulated with mitomycin C-treated HCG-27 cells were transferred to the lower Transwell chamber. After 3 and 5 hr of incubation, the number of reactive T cells that had migrated to the lower chamber due to chemotaxis were determined. More cells had migrated to the lower chamber in the 15×19 CAR group compared to the NT and conventional CAR groups (**Figure 4D**). Additionally, without any changes to other conditions, the NT, conventional CAR, and 15×19 CAR cells were incubated with the anti-CD215 antibody, anti-CCR7 antibody, or isotype control for 3 hr. The blockade of CCL19/CCR7 signaling with the anti-CCR7 antibody resulted in a remarkable decline in the chemotactic ability of 15×19 CAR cells (**Figure 4E**). These data indicate that IL-15 and CCL19 secreted by 15×19 CAR T cells promote T cell proliferation and migration, respectively.

### Rapid and enhanced killing ability of 15×19 CAR T cells

**Figure 5:**
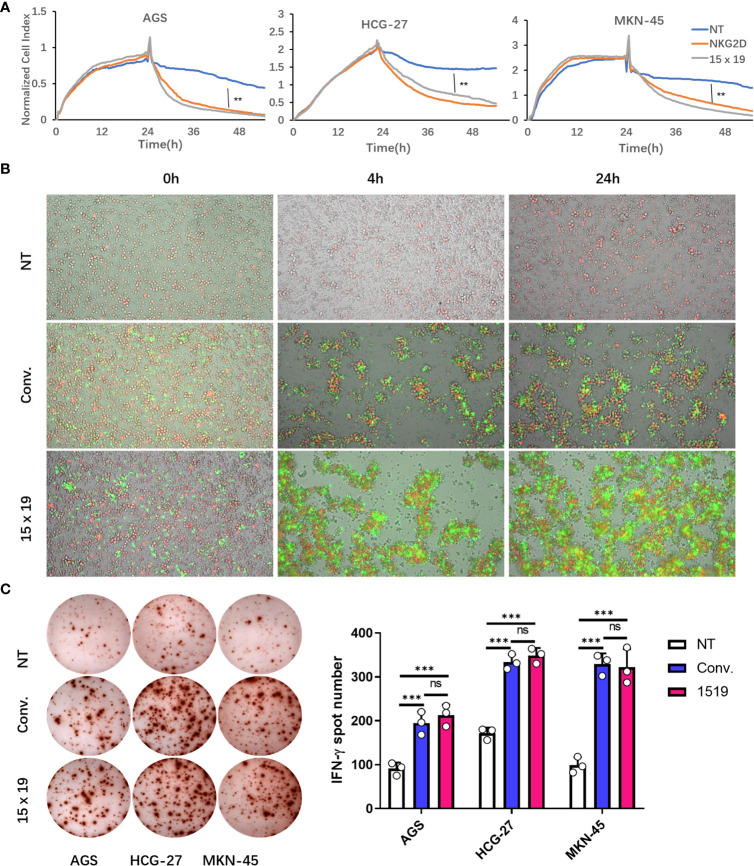
15 × 19 CAR T cells enhance cytotoxicity and IFN-γ production. **(A)**, The cell index values are recorded following co-culture of cells in the NT, conventional CAR and 15 × 19 CAR groups with gastric cancer cells at a ratio of 1:1 for 24 hours using the xCELLigence system. Gastric cancer cells are more effectively killed within 24 hours in the conventional CAR and 15 × 19 CAR groups than in the NT group (*P* < 0.01), while no significant difference was seen between conventional CAR and 15 × 19 CAR groups (*P* > 0.05); **(B)**, Dynamic changes in the fluorescent intensity following co-culture of cells in the NT, conventional CAR and 15 × 19 CAR groups with gastric cancer cells at a ratio of 1:1 at 0, 4 and 24 hours. There are plenty of gastric cancer cells (red fluorescence) adherent to the plate wall 24 hour post-incubation in the NT group, while gastric cancer cells (red fluorescence) are almost completely killed in the conventional CAR and 15 × 19 CAR groups within 4 hours post-incubation, together with the expansion of effector cells. A higher degree of expansion of 15 × 19 CAR T cells (green fluorescence) is observed than that of conventional CAR T cells; **(C)**, ELISPOT assay shows lower numbers of IFN-γ spot-forming cells in the NT group than in conventional CAR and 15 × 19 CAR groups (*P* < 0.001), while no significant difference was seen between conventional CAR and 15 × 19 CAR groups (*P* > 0.05). Analyzed for statistical significance by one-way ANOVA with multiple comparisons test. *ns*, no significant difference; **P < 0.01; ***P < 0.001.

The unique tumor cell culture system xCELLigence that employs electrical impedance measurements for RTCA was used to test the antitumor activity of cells in the NT, conventional CAR, and 15×19 CAR groups. RTCA revealed that gastric cancer cells were more effectively killed within 24 hours in the conventional CAR and 15×19 CAR groups compared to the NT group, while no significant difference was seen between the conventional CAR and 15×19 CAR groups (**Figure 5A**). In addition, a low cytotoxic effect was observed in the NT group, as a substantial number of gastric cancer cells remained adherent to the plate 24 hr post-incubation, while almost all gastric cancer cells were killed in the conventional CAR and 15×19 CAR groups within 4 hr post-incubation (**Figure 5B**). Regarding the expansion of effector cells, there was a higher degree of expansion observed with 15×19 CAR T cells compared to conventional CAR T cells, which was in agreement with the findings presented in **Figure 4B**, indicating a massive expansion of 15×19 CAR T cells during a short period of time upon exposure to tumor antigens. The ELISPOT assay showed lower numbers of IFN-γ spot-forming cells in the NT group compared to conventional CAR and 15×19 CAR groups, while no significant difference was seen between conventional CAR and 15×19 CAR groups (**Figure 5C**). Taken together, these data demonstrate that 15×19 CAR T cells can rapidly kill gastric cancer cells, which is accompanied by a massive expansion of the T cells themselves.

### Generation of anti-human NKG2DL CAR T cells producing IL-15 and CCL19

**Figure 6:**
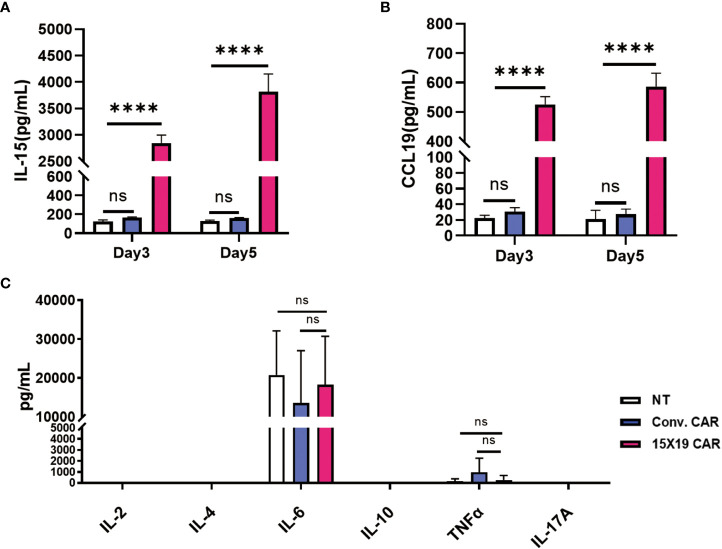
15 × 19 CAR T cells promote IL-15 and CCL19 secretions. Cells in the NT, conventional CAR and 15 × 19 CAR groups are co-cultured with mitomycin C-treated HCG-27 cells at a ratio of 1:1 for 3 or 5 days, and the IL-15 and CCL19 levels are detected in the culture supernatant using ELISA assay. **(A)**, A higher IL-15 level is detected in the 15 × 19 CAR group than in the NT and conventional CAR groups; **(B)**, A higher CCR19 level is detected in the 15**×** 19 CAR group than in the NT and conventional CAR groups; **(C)**, Following co-culture of effector cells and mitomycin C-treated HCG-27 cells at a ratio of 1:1 for 5 days, the greatest IL-6 level is detected; however, no significant difference is seen among the three groups (*P* > 0.05). Low IL-2, IL-4, IL-10, TNF-α and IL-17A levels are detected, with no significant difference detected among the three groups (*P* > 0.05). Representative data are captured from three independent experiments. One-way ANOVA with multiple comparisons test. *ns*, no significant difference; ****P < 0.000,1.

To analyze the function of 15×19 CAR T cells, HCG-27 cells were co-incubated with effector cells at a ratio of 1:1 for 3 or 5 days, and the culture supernatant was harvested. 15×19 CAR T cells were found to significantly promote IL-15 (**Figure 6A**) and CCL19 secretions in the supernatant (**Figure 6B**) as measured by ELISA, while only low levels of IL-2, IL-4, IL-10, TNF-α and IL-17A were detected in the 15×19 CAR group (**Figure 6C**). Although high levels of IL-6 were secreted, the number of 15×19 CAR T cells was not higher compared to the conventional CAR or NT groups (**Figure 6C**). These results demonstrated that the 15×19 CAR T cells secrete large volumes of IL-15 and CCL19 upon stimulation with tumor antigens.

### 15×19 CAR T cells mediate cytotoxicity to cancer in situ

**Figure 7:**
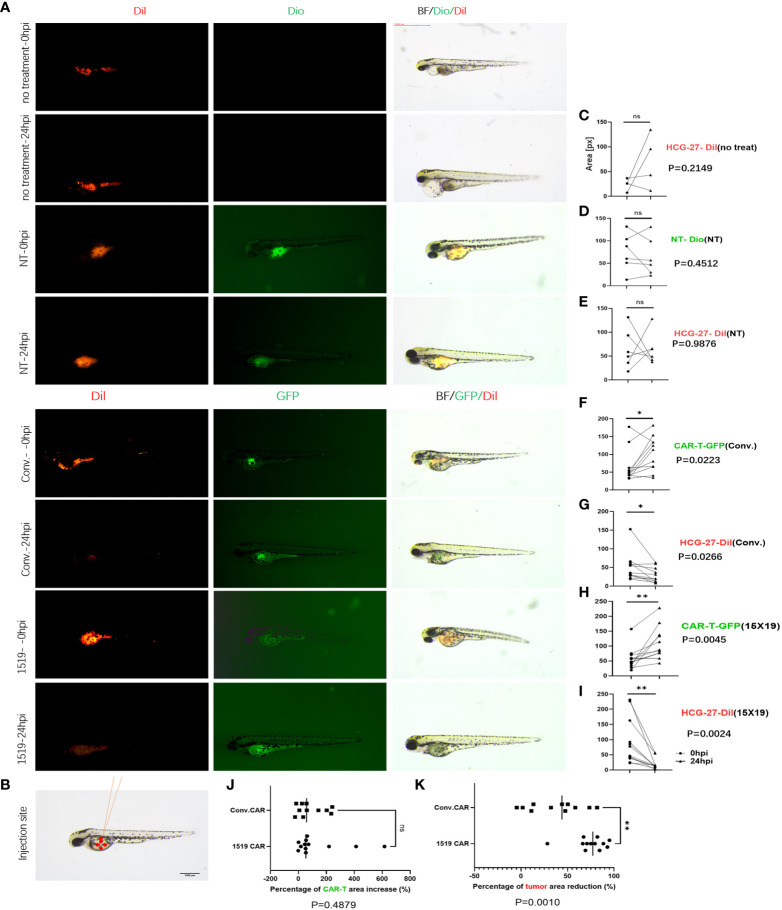
15×19 CAR T cells mediate cancer cells oncolysis in Zebrafish. **(A)**, Cell fluorescence is visualized using fluorescent stereomicroscopy 0 (0 hpi) and 24 (24 hpi) hour post-infection,; **(B)**, A diagrammatic sketch of tumor cells and effector cells injection into the vitellicle, scale bar = 1000 μM. **(C)**, No effector cells are injected in the untreated group, without green fluorescence seen, while no reduction is seen in the area of fluorescence in HCG-27 cells (red fluorescence); **(D)**, No apparent expansion of T cells (green fluorescence) is seen in the NT group 24 hour post-infection; **(E)**, No reduction in the area of fluorescence in HCG-27 cells 24 hours post-infection; **(F)**, Partial expansion of cells is seen in conventional CAR 24 hours post-infection; **(G)**, A reduction is observed in the area of fluorescence in HCG-27 cells in conventional CAR group 24 hours post-infection; **(H)**, Apparent expansion of 15 × 19 CAR T cells is found in the 15 × 19 CAR group 24 hours post-infection; **(I)**, A significant reduction is observed in the area of fluorescence in HCG-27 cells in the 15 × 19 CAR group24 hours post-infection. (**J**), There is no significant difference between the amplification of 15 × 19 CAR T cells and conventional CAR T cells; (**K**), A higher reduction is seen in the HCG-27 cells in the 15 × 19 CAR group. Analyzed for statistical significance by paired Student’s *t* test. ns, no significant difference; *P < 0.05; **P < 0.01.

Previous studies have shown successful xenotransplantation of pancreatic cancer, ovarian cancer, gliomas, breast cancer, prostate cancer, Ewing’s sarcoma and leukemia cell lines into zebrafish embryos, and zebrafish xenotransplantation models have been used for screening of antitumor chemicals (33–35). In the current study, the zebrafish xenotransplantation model was employed to examine the *in vivo* cytotoxicity of CAR T cells against primary gastric cancer, and to visualize the cytotoxicity of CAR T cells against HCG-27 cells. HCG-27 cells (approximately 200) were injected into the vitellicle of each zebrafish and the same number of effector cells were injected into the same site 24 hours post-injection, which was not combined with IL-2. Fluorescent stereomicroscopy was used to monitor the survival of gastric cancer cells and effector cells at 0 and 24 hr (**Figure 7A**). HCG-27 cells were stained with Dil and cells in the NT group were stained with DiO, while cells in the conventional CAR and 15×19 CAR groups expressed GFP. The area of fluorescence in each zebrafish was estimated using ImageJ software.

Cancer cells and immune cells were injected into the vitellicle (**Figure 7B**). The area of HCG-27 fluorescence showed no reduction in the untreated group, and even trended toward an increase (**Figure 7C**). There was no reduction in the area of fluorescence in the NT group (**Figure 7E**), and no expansion of effector T cells (**Figure 7D**). However, an expansion of conventional CAR T cells (*P* = 0.023, 3 vs. 24 hpi) (**Figure 7F**) and 15×19 CAR T cells (*P* = 0.004, 5 vs. 24 hpi) (**Figure 7H**) was observed following stimulation with HCG-27 cells, but there was no significant difference in the amplification ratio between 15×19 CAR and conventional CAR T cells (*P* = 0.4879) (**Figure 7J**). In addition, the area of HCG-27 fluorescence was reduced in the conventional CAR (*P* = 0.0266) (**Figure 7G**) and 15×19 CAR groups (*P* = 0.0024) (**Figure 7I**) relative to 24 hpi, with a higher reduction seen in the 15×19 CAR group (*P* = 0.0010) (**Figure 7K**).

**Figure 8:**
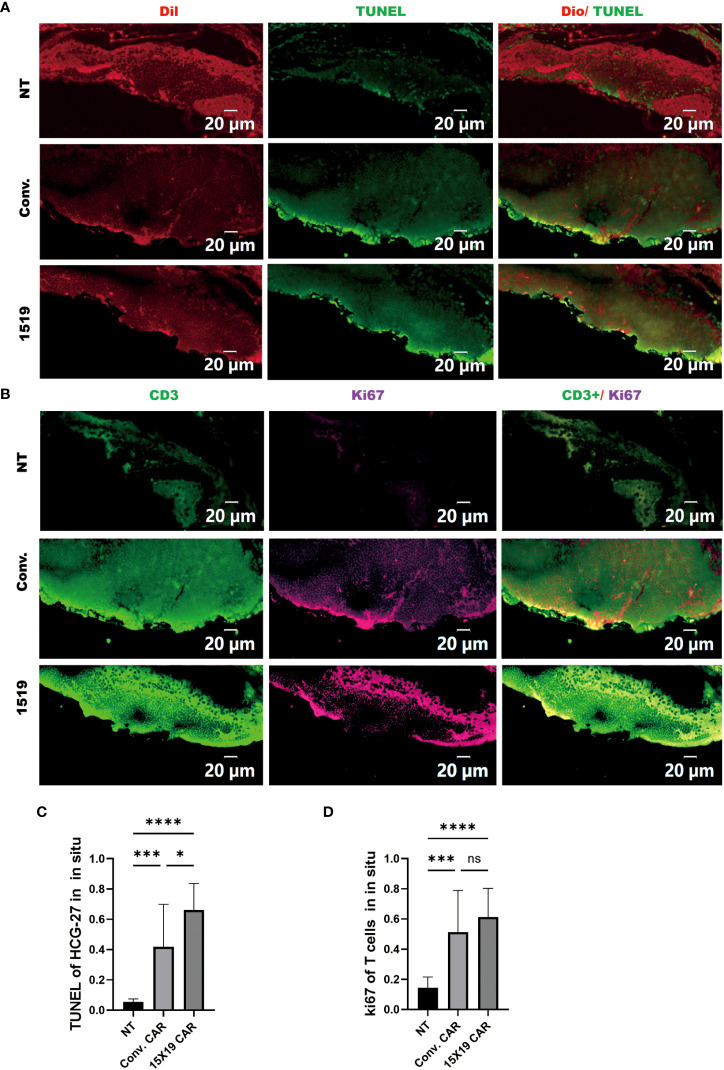
Proliferation of transplanted T cells and tumor apoptosis *in situ* in Zebrafish. Zebrafish were frozen and serially sectioned, and TUNEL immunofluorescence was used to detect tumor apoptosis, and Ki67 to detect T cell proliferation. **(A)**, Representative images of tumor apoptosis (TUNEL : DIO); **(B)** representative images of proliferating T cells (Ki-67:CD3); **(C)** percentage of TUNEL-positive cells in HCG-27 cells; **(D)**, percentage of Ki-67-positive cells in T cells. Data are representative images of > 3 independent, reproducible experiments. Statistical significance analysis was performed using one-way ANOVA and multiple comparison tests. ns, Differences are not significant;*p < 0.05;***p < 0.001;****p < 0.0001, scale bar = 25 μM.

Subsequently, apoptosis of HGC-27 target cells in zebrafish embryos was confirmed 4 days after the T-cell injection in zebrafish using a TUNEL assay performed on frozen sections. We found that both 15×19 CAR-T cells and conventional CAR-T cells induced apoptosis of HCG cells *in vivo* at a higher rate than NT cells. In addition, the 15×19 CAR-T cell group induced higher levels of apoptosis in tumor cells than the conventional CAR-T group. (**Figures 8A, C**). To measure T cell proliferation *in vivo*, Ki-67 was used as a human-specific proliferation marker (no cross-reactivity with zebrafish). T-cell proliferation was significantly higher in the 15×19 CAR group compared to the NT groups (**Figures 8B, D**). Therefore, the zebrafish xenograft assay demonstrated the *in situ* cytotoxicity of 15×19 CAR T cells against gastric cancer and T cell expansion ability.

### Elimination of metastatic cancer cells by 15×19 CAR T cells

Metastasis is the most common cause of cancer-related mortality. Clinically visible metastasis is the final stage of cancer metastasis derived from an abundance of cancer cells. If a primary tumor contains several hundred cancer cells, microscopic metastasis may occur during the very early stages, but it is not detectable even with state-of-the-art imaging analysis systems in patients or mammalian cancer models. The zebrafish xenograft model allows for visualization of microscopic single-cell metastasis or very small metastatic nodules.

**Figure 9:**
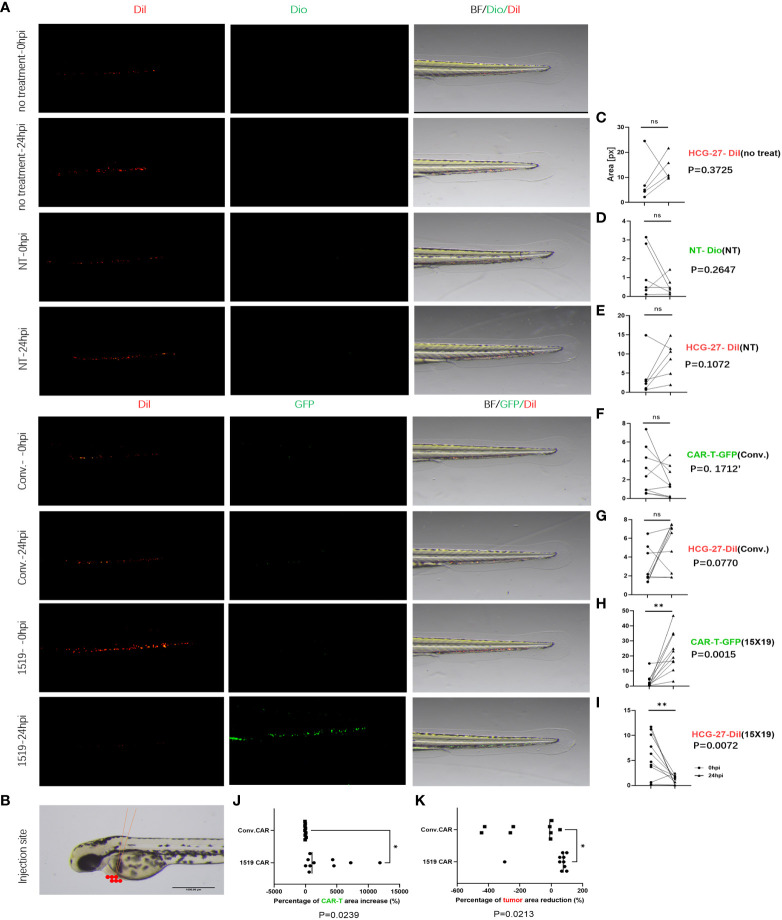
Elimination of metastatic cancer cells by 15×19 CAR T cells. **(A)** Zebrafish model of metastasis. Gastric cancer HCG-27 cells (red) are injected into the perivitelline space of zebrafish to induce extensive cancer metastasis in zebrafish. Then, immune cells (green) are injected into the same site. Cell staining and the time points of observation are the same with the *in situ* cancer; **(B)**, A diagrammatic sketch of tumor cells and effector cells injection into the perivitelline space, scale bar = 1000 μM; **(C)**, No shrinking of the metastatic tumors in the untreated group 24 hours post-injection; **(D)**, NT cells rarely reach the metastatic cells in the NT group, and the cell numbers do not increase 24 hours post-injection; **(E)**, No reduction is seen in the number of metastatic tumors in the NT group 24 hours post-injection; **(F)**, conv.CAR T cells rarely reach the metastatic cells in the conv.CAR T group, and the cell numbers do not increase 24 hours post-injection; **(G)**, No reduction is seen in the number of metastatic tumors in the conv.CAR T group 24 hours post-injection; **(H)**, A large number of 15 × 19 CAR T cells reach the metastatic site and amplify greatly 24 hours post-injection; **(I)**, A significant reduction is seen in the number of metastatic tumors in the 15 × 19 CAR T group 24 hours post-injection. **(J)**, Significantly more 15×19 CAR T cells than conv.CAR T cells; **(K)**, A higher reduction is seen in the number of metastatic cancer cells in the 15 × 19 CAR T group than in the conv.CAR T. Analyzed for statistical significance by paired Student’s t test. *P < 0.05; **P < 0.01.

The cytotoxicity of 15×19 CAR T cells was tested against metastatic cancer cells using the zebrafish model. The cell numbers and time of injections were the same as the *in situ* tumor model, but the injection sites were different (**Figure 9A**). HCG-27 cells were injected into the perivitelline space (**Figure 9B**), and the xenograft tumor predominantly occurred in the zebrafish tail and body. There was no significant reduction of metastatic cancer cells seen in untreated (**Figure 9C**), NT (**Figure 9E**), or conventional CAR T cells groups (**Figure 9G**), and some NT (**Figure 9D**) or conventional CAR T cells (**Figure 9F**) were present at metastatic sites. After 24 hours, a remarkable reduction was seen in the number of 15×19 CAR T cells-derived metastatic cancer cells (**Figure 9I**), and 15×19 CAR T cells had migrated to metastatic sites and amplified considerably (**Figure 9H**). The amplification of 15×19 CAR T cells was significantly higher compared to conventional CAR T cells (*P* = 0.0239) (**Figure 9J**). The reduction in metastatic tumor cells was significantly greater in the 15×19 CAR group compared to the conventional CAR group (*P* = 0.0213) (**Figure 9K**). When 15×19 CAR T cells were treated with an anti-CCR7 antibody, their cytotoxicity against metastatic tumors was reduced and the number of cells did not increase (**Supplementary Figure S2**).

**Figure 10:**
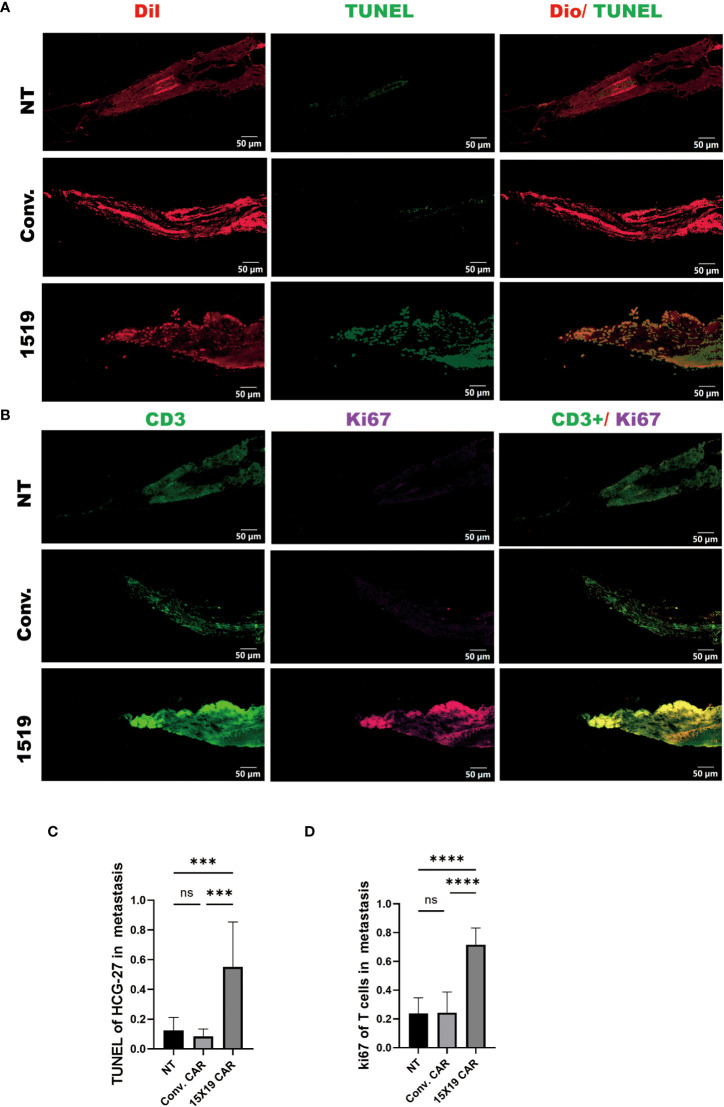
Proliferation of transplanted T cells and tumor apoptosis in zebrafish metastases. Immunofluorescence of TUNEL and ki-67 were performed on transplanted tumor cells and T cells separately. **(A)**, Representative images of tumor apoptosis (TUNEL : DIO); **(B)**, representative images of proliferating T cells (Ki-67:CD3). **(C)**, Proportion of TUNEL-positive cells in HCG-27 cells; **(D)**, Proportion of Ki-67-positive cells in T cells. Statistical significance analysis was performed using one-way ANOVA and multiple comparison test. ns, Differences are not significant; ***p < 0.001; ****p < 0.0001, scale bar = 50 μM.

To determine the anti-tumor activity of 15×19 CAR T cells at a metastatic site, immunofluorescence was used to observe the apoptosis using TUNEL staining of tumor cells and the proliferation of T cells using Ki-67 at 4 dpi. The results showed that the apoptosis of metastatic tumor cells in the 15×19 CAR T cells group was significantly higher compared to the other groups (**Figures 10A, C**), and the proliferation of T cells was also significantly higher (**Figures 10B, D**). These findings demonstrate that 15×19 CAR T cells effectively eliminated metastatic cancer cells in zebrafish.

## Discussion

In the current study, IL-15 and CCL19 were introduced to NKG2D CAR T cells to generate novel NKG2D 15×19 CAR T cells. Compared to conventional CAR T cells, NKG2D 15×19 CAR T cells recognized eight NKG2DLs expressed on the surface of most solid tumors. This allows the cells to target a wide variety of cancers, as well as diverse ligands on the same tumor cell, which can reduce off-target effects. In addition, NKG2DLs are not expressed by healthy cells, therefore few adverse effects are seen. The novel NKG2D 15×19 CAR T cells generated expressed both the IL-15 cytokine and CCL19 chemokine, allowing for long-term survival and persistent effects of CAR T memory cells. This also allows for increased infiltration of immune cells into cancer sites, thereby increasing their effectiveness to target tumors.

To date, four generations of CAR T cells have been generated. The first-generation only contains the signaling molecule CD3ζ. The second-generation contains one co-stimulatory molecule based on the first-generation of CAR T cells, and includes IgG superfamily member CD28 as well as TNF superfamily members CD40L, OX-40 and 4-1BB. The third-generation of CAR T cells contains two co-stimulatory domains (36), and fourth-generation cells express additional signals, including cytokines and chemokines (37). Currently, clinical trials predominantly focus on the second CAR T cells. However, these cells have low rates of survival, infiltration, and lasting effects. Therefore, third-generation NKG2D CAR T cells were generated in the current study, and the addition of IL-15 and CCL19 resulted in fourth-generation CAR T cells with the goal of improving penetration, proliferation, survival, and tumor-killing abilities of CAR T cells in immunosuppressive tumor microenvironments.

The ability of CAR T cells to influence gastric cancer has been extensively investigated, and includes research on epithelial growth factor receptor (EGFR) (38), epithelial cell adhesion molecule (EpCAM) (39), and mesothelin (40), and has also focused on NKG2D (41). Recently, results from an open-label, single-arm, phase 1 clinical trial of claudin (CLDN)18.2-targeted CAR T cells (CT041) showed that CT041 has promising efficacy with an acceptable safety profile in patients with heavily pretreated, CLDN18.2-positive digestive system cancers, particularly in those with gastric cancer (42). The treatments for solid tumors are inherently more challenging and difficult compared to hematological malignancies due to the heterogeneity of solid tumor antigens, the immune suppression of solid tumor microenvironments, and the exhaustion of tumor-infiltrating T lymphocytes (43). The development of novel CAR T cells able to target solid tumors is ongoing, and the addition of cytokines into CAR T cells has been proposed to overcome these obstacles. Engineered modifications to cytotoxic T cells was found to allow the release of inducible IL-12 during the entry of CAR into tumor foci, which destroyed the cancer cells that had escaped antigen-dependent killing (44). Introduction of IL-7 into CAR T cells was shown to increase antitumor activity compared to conventional CAR T cells, and the generated CAR T cells elicited memory responses to tumors (45).

Unlike conventional CAR T cell therapy, NKG2D CAR T cells do not contain antibody fragments that recognize tumor surface antigens or protein structures that trigger the immune response, which reduces the likelihood of CAR T cell rejection by the patient’s immune system (46, 47). Previous studies have shown that tumor-derived immunosuppressive cells, such as myeloid-derived suppressor cells (MDSCs) or regulatory T (Treg) cells, also express NKG2DLs (48, 49). NKG2D CAR T cells may target immunosuppressive cells and partly reverse immunosuppressive tumor microenvironments, thereby increasing antitumor immune responses. In addition, NKG2DLs are expressed by endothelial cells within new blood vessels that supply tumors. Therefore, NKG2D CAR T cells may suppress tumor progression through the inhibition of angiogenesis (50). Taken together, NKG2D CAR T cells are remarkably superior to conventional CAR T cells for cancer therapy in terms of safety and efficacy.

Tao et al. first reported that NKG2D-based CAR T cells have potent *in vivo* and *in vitro* anti-tumor activities against gastric cancer, which could be enhanced by the addition of cisplatin (41). Suppressor cells and soluble factors may suppress the persistent antitumor activity of CAR T cells in tumor microenvironments, while hypoxia and a lack of nutrients at the interior of the tumor result in reduced long-term survival and limited expansion of CAR T cells (51, 52). Following introduction of IL-15 to the CAR-19 construct, the CAR T cells developed long-term persistence with a memory stem-cell phenotype, and produced high levels of IL-15 upon T-cell activation, thereby mediating local environments and assisting CAR T cell functions (53). The data within this study revealed that 15×19 CAR T cells were primarily T_cm_ (CD62L^+^CD45RO^+^) cells. In the lymph node, T_cm_ cells are more likely to receive antigen information presented by antigen-presenting cells (APCs) to undergo second activation (54). Our data showed that 15×19 CAR T cells, even with low numbers compared to target cells, retained the capacity to efficiently remove target cells. The tumor cells stimulated the proliferation of 15×19 CAR T cells, up-regulate the expression of activators (CD25 and HLA-DR), and down-regulation the expression of suppressors (CTLA-4 and PD1).

Currently, the efficacy of CAR T cell therapy for the treatment of gastrointestinal tumors remains unsatisfactory, and a major difficulty is the ability of effector cells to reach the tumor site (11). The physical and immune barriers created by the peri-tumor matrix and immune cells prevent efficient infiltration of CAR T cells into tumor tissues (13, 55, 56). CCL19, secreted by fibroblastic reticular cells, are effective for the chemotaxis of peripheral DCs and T cells to reach lymphatic organs (45). In this study, CCL19 was introduced to the NKG2D CAR construct to generate 15×19 CAR T cells, which secreted high levels of CCL19 following recognition of tumor antigens. The increased CCL19 recruited T cells, which was consistent with previous findings (45). In addition, 15×19 CAR T cells did not produce IL-4, IL-10, or IL-17A cytokines upon stimulation by gastric cancer cells. IL-6 is of central importance in the induction of cytokine release syndrome (CRS), and CRS represents a frequent complication of CAR T therapy (57). Tocilizumab, a humanized anti-human IL-6 receptor antibody, has been shown to prevent CAR T cell-mediated CRS (58). Of course, although we did not observe IL-6-driven effects in our studies, this does not exclude the possibility that IL-6 may cause CRS effects in CAR-T cells in a more clinicalsituation.

Currently, the mouse xenotransplantation model is the gold standard for preclinical assessment of CAR T cell therapy (59). However, the process of mouse xenotransplantation can be slow and expensive (60). An additional vertebrate model is therefore important to overcome the shortages of mouse xenotransplantation models. Since there is a lack of a functional adaptive immune system in zebrafish embryos and during early stages of development, any cells transplanted will not be rejected by the immune system (61). This model may achieve single-cell visualization of human cancer cells in zebrafish, allow for quantitative study on primary tumor growth, and investigate early cancer metastasis from primary tumors. Embryonic zebrafish xenograft models have been previously used to investigate CAR T cells *in vivo* (62). In the current study, the cytotoxicity of 15×19 CAR T cells against human gastric cancer cells was evaluated using this *in situ* xenograft model, and 15×19 CAR T cells showed significantly more cytotoxicity against human gastric cancer HCG-27 cells compared to the NT and conventional CAR groups. In the xenograft models, many 15×19 CAR T cells were recruited to metastatic cancer sites where they amplified to effectively kill metastatic tumor cells.

Although zebrafish xenotransplantation can allow for the tracing of individual metastatic cancer cells *in vivo* and the visualization of immune cell-mediated eradication of metastatic cancer cells, there are still shortcomings associated with these experiments compared to mice xenotransplantation. Firstly, zebrafish xenograft experiments were performed below 37°C. At this temperature, the transplanted human cells do not proliferate at the same rate, while immune-compromised mice have the same temperature as humans. Secondly, the cytotoxicity of T cells to tumor cells in zebrafish was observed over a short period. The overall survival (OS) of zebrafish was not assessed, which could be achieved in mice. Thirdly, due to the limited time of the *in vivo* experiments, stromal tumor cells were not formed, and the effect of the tumor microenvironment on the killing of tumor cells by T cells could not be observed. Lastly, although Erica et al. (63) proposed an efficient dissociation protocol for the generation of a single cell suspension from zebrafish embryos and larvae, we were unable to successfully isolate single cells for this experiment. As such, we were unable to detect and analyze the T-cell phenotype in zebrafish *in vivo*.

In summary, the results of the present study demonstrated that the introduction of IL-15 and CCL19 into conventional CAR T cells may enhance the antitumor potential of CAR T cells. Since loss or mutation of target molecules is a mechanism underlying resistance to CAR T cell therapy in cancers, 15×19 CAR T cells may serve as effector cells against tumors and as cell vectors to transfer immunomodulatory molecules to tumor microenvironments. This method can trigger antitumor immune responses, induce persistent cell expansion, increase cytotoxicity, introduce additional cytokines, and reduce T-cell exhaustion following exposure to tumor antigens.

## Data availability statement

The original contributions presented in the study are included in the article/**Supplementary Material**. Further inquiries can be directed to the corresponding authors.

## Ethics statement

The studies involving human participants were reviewed and approved by Ethics Committee of Fujian Cancer Hospital(SQ2019-006-01). The patients/participants provided their written informed consent to participate in this study. The animal study was reviewed and approved by Animal Ethics Committee of Fujian Medical University(FJMU IACUC2021-0455).

## Author contributions

YY conceived and designed the study. ZZ, JL, WL, JH, SC, LW, and MC performed the experiments. ZZ and JH analyzed and interpreted the data. ZZ, SC, and MC provided the first version of the manuscript. ZZ, JL, WL, JH, SC, and MC and YY provided critical comments on revision of the manuscript. YY revised and finalized the manuscript. All authors contributed to the article and approved the submitted version.
