# Supplementary material for: Interleukin-15 and chemokine ligand 19 enhance cytotoxic effects of chimeric antigen receptor T cells using zebrafish xenograft model of gastric cancer
Source: Front Immunol. 2022 Dec 23;13:1002361. doi: 10.3389/fimmu.2022.1002361 (PMC9816141; doi:10.3389/fimmu.2022.1002361)
Supplement: Supplementary file 1 [file DataSheet_1.docx]

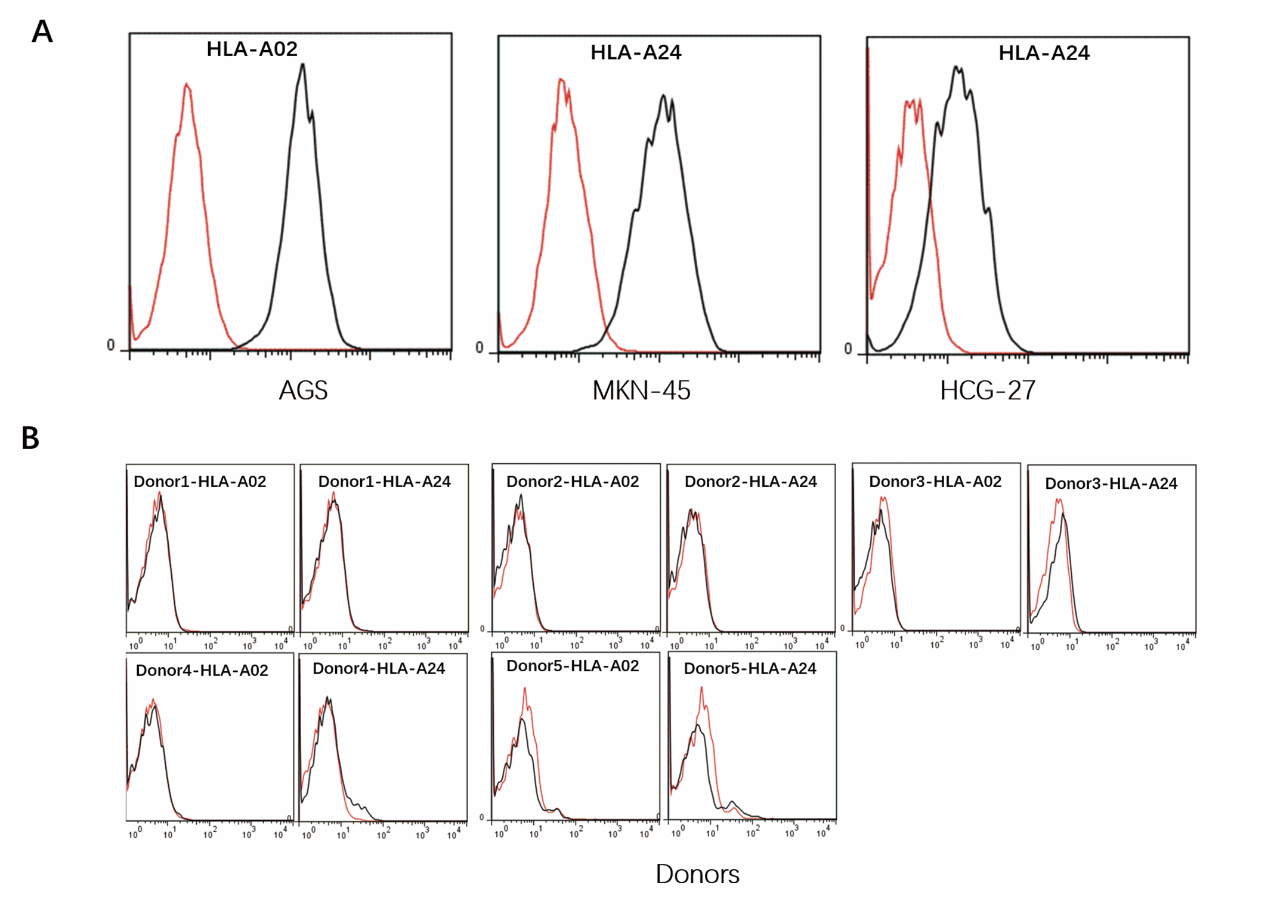


**FIGURE s1. Expression of HLA-A02 and HLA-A02 in human gastric cancer cell lines.**  Flow cytometry data were representative of three independent experiments, The red histogram indicates the expression of the isotype control and the black histogram indicates expression of HLA-A02 or HLA-A02. **(A)** HLA-A02 expression in AGS cell line, HLA-A24 expression MKN-45 and HCG-27; **(B)** HLA-A02 or HLA-A24 expression in none of Donor.


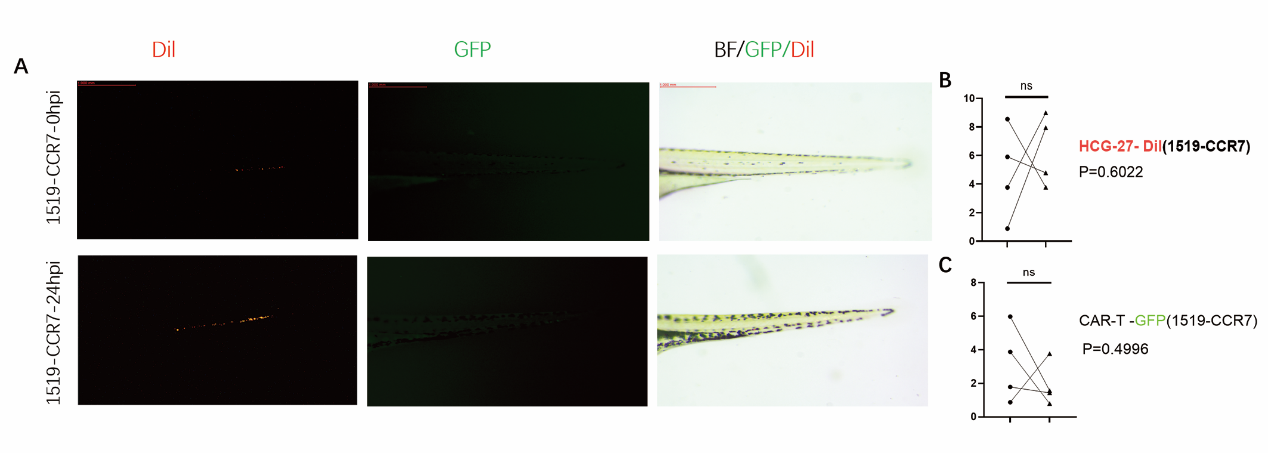


**FIGURE s2. Effect on metastases after blocking 15×19 CAR-T cells with antibody CCR7. (A)**, gastric cancer HCG-27 cells (red) are injected into the perivitelline space of zebrafish to induce extensive cancer metastasis in zebrafish. Then, CCR7 -blocked 15×19 CAR-T cells (green) are injected into the same site. Cell staining and the time points of observation are the same with the in situ cancer; **(B)**, No shrinking of the metastatic tumors 24 hours post-injection; **(C)**, CCR7 -blocked 15×19 CAR-T cells numbers do not increase 24 hours post-injection; Analyzed for statistical significance by paired Student’s t test.
